# Trefoil factor 1 suppresses stemness and enhances chemosensitivity of pancreatic cancer

**DOI:** 10.1002/cam4.7395

**Published:** 2024-06-13

**Authors:** Junpei Yamaguchi, Toshio Kokuryo, Yukihiro Yokoyama, Shunsuke Oishi, Masaki Sunagawa, Takashi Mizuno, Shunsuke Onoe, Nobuyuki Watanabe, Atsushi Ogura, Tomoki Ebata

**Affiliations:** ^1^ Division of Surgical Oncology, Department of Surgery Nagoya University Graduate School of Medicine Nagoya Japan; ^2^ Institute of Transformative Bio‐Molecules Nagoya University Nagoya Japan

**Keywords:** cancer stem cell, chemosensitivity, epithelial‐mesenchymal transition, pancreatic cancer, trefoil factor

## Abstract

**Background and Aims:**

Pancreatic cancer is one of the most lethal malignancies, partly due to resistance to conventional chemotherapy. The chemoresistance of malignant tumors is associated with epithelial‐mesenchymal transition (EMT) and the stemness of cancer cells. The aim of this study is to investigate the availability and functional mechanisms of trefoil factor family 1 (TFF1), a tumor‐suppressive protein in pancreatic carcinogenesis, to treat pancreatic cancer.

**Methods:**

To investigate the role of endogenous TFF1 in human and mice, specimens of human pancreatic cancer and genetically engineered mouse model of pancreatic cancer (KPC/TFF1KO; Pdx1‐Cre/LSL‐KRAS^G12D^/LSL‐p53^R172H^/TFF1^−/−^) were analyzed by immunohistochemistry (IHC). To explore the efficacy of extracellular administration of TFF1, recombinant and chemically synthesized TFF1 were administered to pancreatic cancer cell lines, a xenograft mouse model and a transgenic mouse model.

**Results:**

The deficiency of TFF1 was associated with increased EMT of cancer cells in mouse models of pancreatic cancer, KPC. The expression of TFF1 in cancer cells was associated with better survival rate of the patients who underwent chemotherapy, and loss of TFF1 deteriorated the benefit of gemcitabine in KPC mice. Extracellular administration of TFF1 inhibited gemcitabine‐induced EMT, Wnt pathway activation and cancer stemness, eventually increased apoptosis of pancreatic cancer cells in vitro. In vivo, combined treatment of gemcitabine and subcutaneous administration of TFF1 arrested tumor growth in xenograft mouse model and resulted in the better survival of KPC mice by inhibiting EMT and cancer stemness.

**Conclusion:**

These results indicate that TFF1 can contribute to establishing a novel strategy to treat pancreatic cancer patients by enhancing chemosensitivity.

## Graphical abstract

TFF1 inhibits EMT and stemness in pancreatic cancer and the chemosensitivity of the tumor can be increased by injected TFF1. Combined therapy with TFF1 and traditional chemotherapy could be useful in pancreatic cancer treatment.

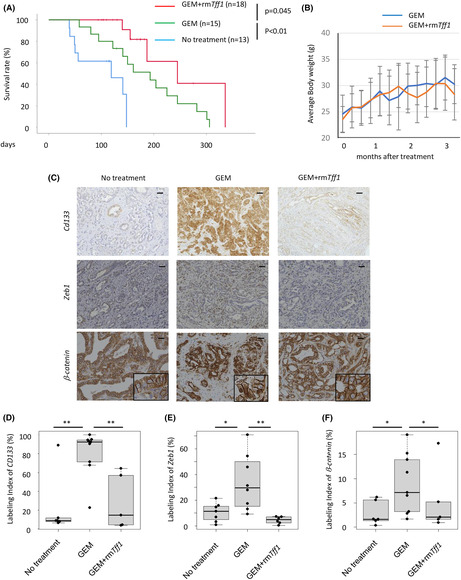

## 1 INTRODUCTION

Although treatments for pancreatic cancer, including surgical resection, irradiation and chemotherapy, have been developed, the efficacy of these treatments and the survival of patients remain undesirable. Tumor chemoresistance hampers the development of treatments. For instance, although multidrug chemotherapy, such as FOLFIRINOX and gemcitabine/nab‐paclitaxel, has contributed to the improvement of patient survival, the incidence of adverse effects is high, and the response rate remains as low as 30%.^1^, ^2^

The mechanisms of chemoresistance have been investigated, and epithelial‐mesenchymal transition (EMT) and cancer stemness were shown to contribute to therapeutic resistance.^3^, ^4^, ^5^, ^6^ EMT is characterized by the conversion of cancer cells from an epithelial phenotype to a mesenchymal phenotype and thus plays a role in various processes during the progression of malignant diseases such as invasion, dissemination to distant organs, and resistance to antitumor drugs. EMT can be triggered by zinc‐finger proteins (such as Snail, Slug, and ZEB1) and various signaling pathways, including the Wnt cascade and PI3A/AKT axis.^7^ Of note, EMT of cancer cells can also be induced by anticancer agents; thus, long‐term chemotherapy results in acquired drug resistance of the tumor.^8^ In addition, EMT has been associated with stemness,^9^, ^10^ and cancer stem cells (CSCs) also show antitherapeutic effects. Given this evidence, treatments that inhibit EMT and the stemness of cancer cells could be an attractive approach.

TFF1 is a secreted protein that exhibits a tumor‐suppressive role in pancreatic carcinogenesis by inhibiting malignant transformation of premalignant lesions, such as pancreatic intraepithelial neoplasms (PanIN).^11^ Interestingly, TFF1 is expressed in the progenitor‐cell niche of the pancreatic epithelium,^12^ suggesting a possible association between TFF1 and stem cells. Here, we show that TFF1 can inhibit EMT and stemness, thus enhancing the chemosensitivity of pancreatic cancer. These results could provide a fundamental basis for novel cancer treatments that could have a significant effect on disease outcome.

## 2 MATERIALS AND METHODS

### 2.1 Human samples

Human pancreatic samples were obtained from the surgically resected pancreatic cancer specimen (*n* = 91) at Nagoya University Hospital. Among these patients, the survival of those who suffered postoperative recurrence of the disease and received chemotherapy (*n* = 46) was analyzed.

### 2.2 Histology and immunohistochemistry

The methods for IHC are described in our previous report.^11^ In short, the sections were incubated overnight at 4°С with each primary antibody (Table S1). Then the specimens were incubated at room temperature with secondary antibodies for 1 h. Next, proteins were stained by EnVision Detection Systems (Dako Japan, Tokyo, Japan). Counterstain was performed with hematoxylin. Five to ten areas were randomly imaged and analyzed. The labeling index (percentage of positive cells) was calculated by the number of positive cells divided by the number of all cancer cells.

### 2.3 Cell culture

The methods for cell culture are described in our previous report.^11^ In short, all cell lines were maintained in RPMI medium (Invitrogen Life Technologies, Carlsbad, CA) containing 5% fetal bovine serum (Equitech‐Bio, Inc., Kereville, TX).

### 2.4 Transfection of TFF1 overexpression plasmid

The plasmids for the overexpression of TFF1 was transfected into pancreatic cancer cell lines using a CUY21EDIT (BEX, Tokyo, Japan) according to the manufacturer's protocol. Cancer cell lines were maintained for 48 h after the transfection and then analyzed.

### 2.5 The synthesis of human TFF1

Synthetic human TFF1 (shTFF1) was prepared by automated Fmoc solid phase peptide synthesis using CS136XT (CSbio, Menlo Park, CA) and Wang polystyrene resin (Merck, DE). Fmoc deprotections were performed with 20% piperidine in *N*,*N*‐dimethylformamide (DMF, 2 × 8 min). Coupling was performed with Fmoc‐amino acid (4.0 equiv relative to resin substitution), *O*‐(1H‐6‐chlorobenzotrazol‐1‐yl)‐*N*,*N*,*N′*,*N′*‐tetramethyluronium hexafluorophosphate (3.9 equiv) and *N*‐methylmorpholine (8.0 equiv) in DMF for 60 min. Double coupling was performed with repeated coupling step if necessary. A mixture of 97:1:2 trifluoroacetic acid:triisopropylsilane: H_2_O (15 mL/g resin) was added to the obtained dry resin, and the suspension was shaken for 2 h. The resin was removed by filtration. After volatile removed by reduced pressure, the residue was washed with diethyl ether (ca. 15 mL/g resin). The crude material was purified by reversed‐phase high‐performance liquid chromatography (HPLC) on a Phenomenex Jupiter C18 column (300 Å pore size, 5 μm, 30 mm I.D. × 250 mm). The purified reduced peptide was denatured using denatured buffer. The solution was diluted with a nine‐fold volume of folding buffer containing 0.1 mM phosphate buffer (pH 8.0) with 10% dimethyl sulfoxide and stirred for 24 h at ambient temperature. Refolded shTFF1 was obtained after HPLC purification on a Phenomenex Jupiter C18 column (300 Å pore size, 5 μm, 10 mm I.D. × 250 mm) and lyophilization.

### 2.6 Cell treatment by TFF1 protein

Recombinant human TFF1 (rhTFF1) and mouse TFF1 (rmTFF1) were purchased from Peprotech (Cranbury, NJ). Synthesized human TFF1 (shTFF1) was synthesized and provided by Craftide (Nagoya, Japan). Human pancreatic cancer cell lines were plated in 6‐well plates and then treated with gemcitabine and/or TFF1 (either rhTFF1 or shTFF1) for 36 h and then analyzed.

### 2.7 Cell proliferation assay

The relative cell number was assessed using a WST‐1 cell proliferation assay system (Roche, Indianapolis, IN). The cells were treated and seeded in a 96‐well plate with the concentration of 5 × 10^3^ cells per well. Relative cell numbers were assessed by WST‐1 reagent 72 h after the treatment.

### 2.8 Western blotting

Proteins were obtained from the cells using Laemmli sample buffer (Wako, Japan), subjected to 10% SDS‐polyacrylamide electrophoresis, and electrophoretically transferred onto polyvinylidene difluoride membranes. The primary antibodies for the detection of the protein are listed in Table S2. The membrane was incubated with primary antibodies then secondary antibodies sequentially. Protein expression was visualized using Pierce Western Blot Substrate (Thermo, Rockford, AZ).

### 2.9 Real‐Time PCR

Total RNA was extracted from cultured cells using a QIAcube (Qiagen, Hilden, Germany) and used to synthesize complementary DNA (High Capacity cDNA Reverse Transcription kit, Applied Biosystems, South San Francisco, CA). Real‐time PCR amplification was performed using a 7300 Fast Real‐Time PCR System (Applied Biosystems). The relative expressions of genes were standardized by 18S expression. The TaqMan probes and primers were obtained from Applied Biosystems (listed in Table S3).

### 2.10 Apoptosis assay

Apoptosis of the cells were analyzed by the Muse Annexin V and Dead Cell Kit (Millipore, Darmstadt, Germany). The cells were seeded in 6‐well plates, treated by gemcitabine and/or TFF1, and then rates of apoptotic cells were analyzed.

### 2.11 Flow cytometry

After the cells were rinsed with PBS, cells were incubated with antibodies against CD133 (BD Pharmingen, clone: W6B3C1) and Hoechst 33342 (Invitrogen, Waltham, MA) for 1 h at room temperature. Cells were washed and resuspended with PBS, and then assessed by flow cytometry (FACSCanto 2, BD).

### 2.12 Tumor sphere formation assay

Sphere formation ability was assessed using 3D Tumorsphere Medium XF (PromoCell, Heidelberg, Germany) according to the manufactures' protocol. In short, 40,000 KLM1 cells were seeded in 6‐well plate with ultra‐low attachment surface (CORNING, NY) and incubated for 7 days. Spheres were collected by gravity sedimentation and digested by trypsin–EDTA, then plated again. On day 14, the number and diameter of spheres were calculated.

### 2.13 Animals

Pdx1‐Cre (stock number: 014647), LSL‐p53^R172H^ (stock number: 008652), and LSL‐KRAS^G12D^ (stock number: 008179) mice were purchased from the Jackson Laboratory (Bar Harbor, ME, USA).^13^ TFF1‐KO (Tff1^tm1a(EUCOMM)Wtsi^) mice were purchased from the IMPC (International Mouse Phenotyping Consortium).^14^ Pdx1‐Cre (C), LSL‐p53^R172H^ (P), and LSL‐KRAS^G12D^ (K) mice were bred with TFF1‐KO mice to obtain KPC/TFF1KO mice. Murine recombinant TFF1 (mrTFF1) was purchased from Peprotech (Cranbury, NJ). Gemcitabine (100 mg/kg, intraperitoneal injection, twice per week) and mrTFF1 (1 μg/mouse, subcutaneous injection, twice per week) were administered at the age of 2 months. The mice were sacrificed when they became moribund, and their pancreatic samples were harvested for analysis.

### 2.14 Xenograft mouse model

Eight‐weeks‐old male BALB/c nude mice were purchased from SLC Japan (Nagoya, Japan). Panc1 cells (1 × 10^7^) were implanted into each mouse to generate xenograft mouse model. The tumor length (L) and width (W) were measured and tumor volume was calculated by (L × W^2^)/2.

### 2.15 Statistics

All data are presented as the mean ± SD or as box‐and‐whisker plots (centerline, median; box limits, upper and lower quartiles; whiskers, 1.5× interquartile range; points, outliers) as appropriate. Differences were tested for significance by *T*‐test or Mann–Whitney as appropriate. The survival of humans and mice was calculated using the Kaplan–Meier method. The log‐rank test was employed to analyze the differences. These statistical analyses were performed using publicly available software R or SPSS (Statistical Package for the Social Sciences). Difference with *p* value <0.05 was recognized as statistically significant.

## 3 RESULTS

### 3.1 TFF1 inhibits EMT of pancreatic cancer *in vivo*

**FIGURE 1:**
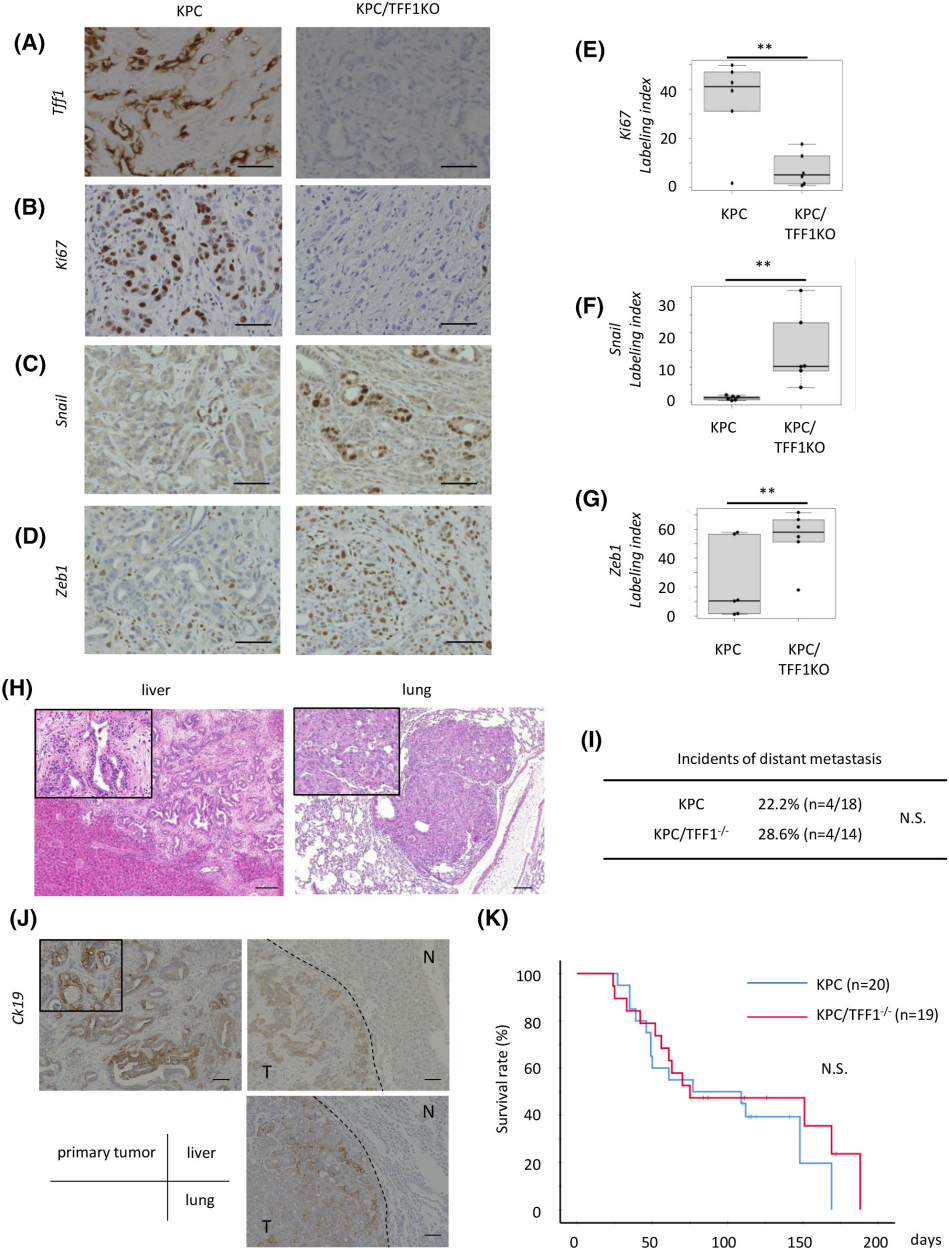
TFF1 inhibits EMT in pancreatic cancer *in vivo*. (A–D) Representative IHC images of TFF1, Ki67, Snail and ZEB1 in PDAC developed in the KPC and KPC/TFF1KO mice. 60 images from 12 mice were investigated and counted for each molecule. Scale bars; 100 μm. (E–G) Quantification of the positive cells shown as the labeling index. **p < 0.01 (H) Representative microscopic images of distant metastasis found in the liver and lung. Scale bars; 200 μm. (I) The incidence of distant metastasis in each mouse model. (J) Representative IHC image of CK19 in each tumor. T; tumor, N; normal tissue. Scale bars; 100 μm. (K) Overall survival of each mouse model.

Although we found that TFF1 functions as a tumor suppressor in pancreatic premalignant lesions, it is unclear whether it has the same function in mature malignant tumors *in vivo*. To clarify this, we employed a mouse model of pancreatic cancer development, KPC mice (Pdx1‐Cre; LSL‐KRAS^G12D^; LSL‐p53^R172H^), which were bred with TFF1KO mice to generate KPC/TFF1KO mice, and the tumor characteristics including EMT were compared between these mouse models. Knockout of TFF1 protein was confirmed (Figure 1A). The proliferative activity (Ki67) of tumor cells was lower in the KPC/TFF1KO mice (Figure 1B,E), whereas the labelling index of the EMT markers Snail (Figure 1C,F) and ZEB1 (Figure 1D,G) was significantly higher in the KPC/TFF1KO mice than the other mice. These results are consistent with the concept that the attenuation of proliferation is one of the hallmarks of EMT.^15^, ^16^ Since EMT is thought to promote invasion and metastasis of pancreatic cancer, the incidence of distant metastasis was then analyzed. Although liver and lung metastases were found and histologically confirmed in some mice (Figure 1H), the incidence of metastasis was similar between the KPC and KPC/TFF1KO mice (Figure 1I). The primary tumor and liver/lung tumor shared similar molecular characteristic (CK19; pancreatic epithelial marker), confirming that they were the metastatic lesion of pancreatic cancer (Figure 1J). In addition, the survival of these mice did not show any difference (Figure 1K). These results seem inconsistent with the traditional understanding of the nature of EMT; however, recent reports have suggested that EMT does not necessarily promote metastasis of the tumor in vivo^5^, ^6^; thus, it appears that our findings are consistent with recent data on EMT.

### 3.2 TFF1 expression enhances the chemosensitivity of pancreatic cancer in humans and mice

**FIGURE 2:**
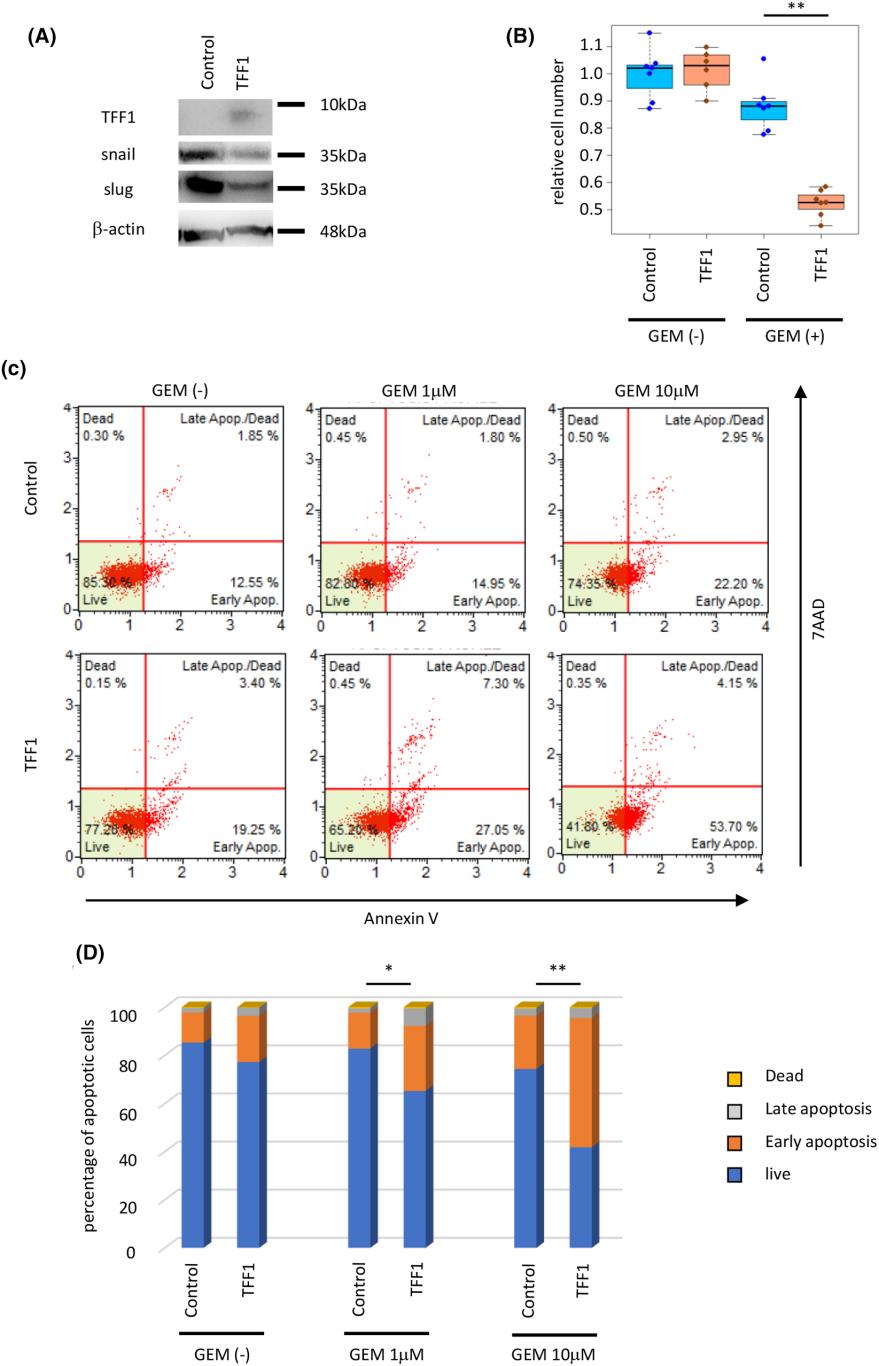
Overexpression of TFF1 suppresses EMT and increases chemosensitivity of pancreatic cancer cells (panc1) *in vitro*. (A) Protein expression of TFF1, Snail, and Slug in the cells transfected with the control and TFF1‐overexpressing plasmids assessed by western blotting. (B) Relative cell number of each transfected cell line treated with gemcitabine (1 μM) for 72 h. **p < 0.01 (C,D) The rate of apoptosis of each group was assessed by the Muse™ Annexin V & Dead Cell Kit. *p < 0.05, **p < 0.01.

Given the above findings, we hypothesized that TFF1 might be associated with the chemosensitivity of pancreatic cancer. We transfected the TFF1 overexpression vector into the pancreatic cancer cell line Panc1. Western blotting confirmed the expression of TFF1 induced by the vector, and EMT markers (Snail and Slug) were found to be suppressed in the TFF1‐expressing Panc1 cells (Figure 2A). The proliferative ability assessed by WST‐1 assays did not show a difference between the control and TFF1‐expressing cells. This result seems contradicts the findings showing the decrease of proliferative ability of cancer cells in TFF1KO mice; this discrepancy between *in vitro* and *in vivo* indicates the potential importance of microenvironment of pancreatic cancer. These cells were then treated with gemcitabine, and the cell number was substantially decreased in the TFF1‐expressing cells (Figure 2B). In addition, the rate of apoptotic cells was higher in the TFF1‐expressing cells than the control cells (Figure 2C,D), confirming that the cellular expression of TFF1 is associated with the chemosensitivity of pancreatic cancer cells.

**FIGURE 3:**
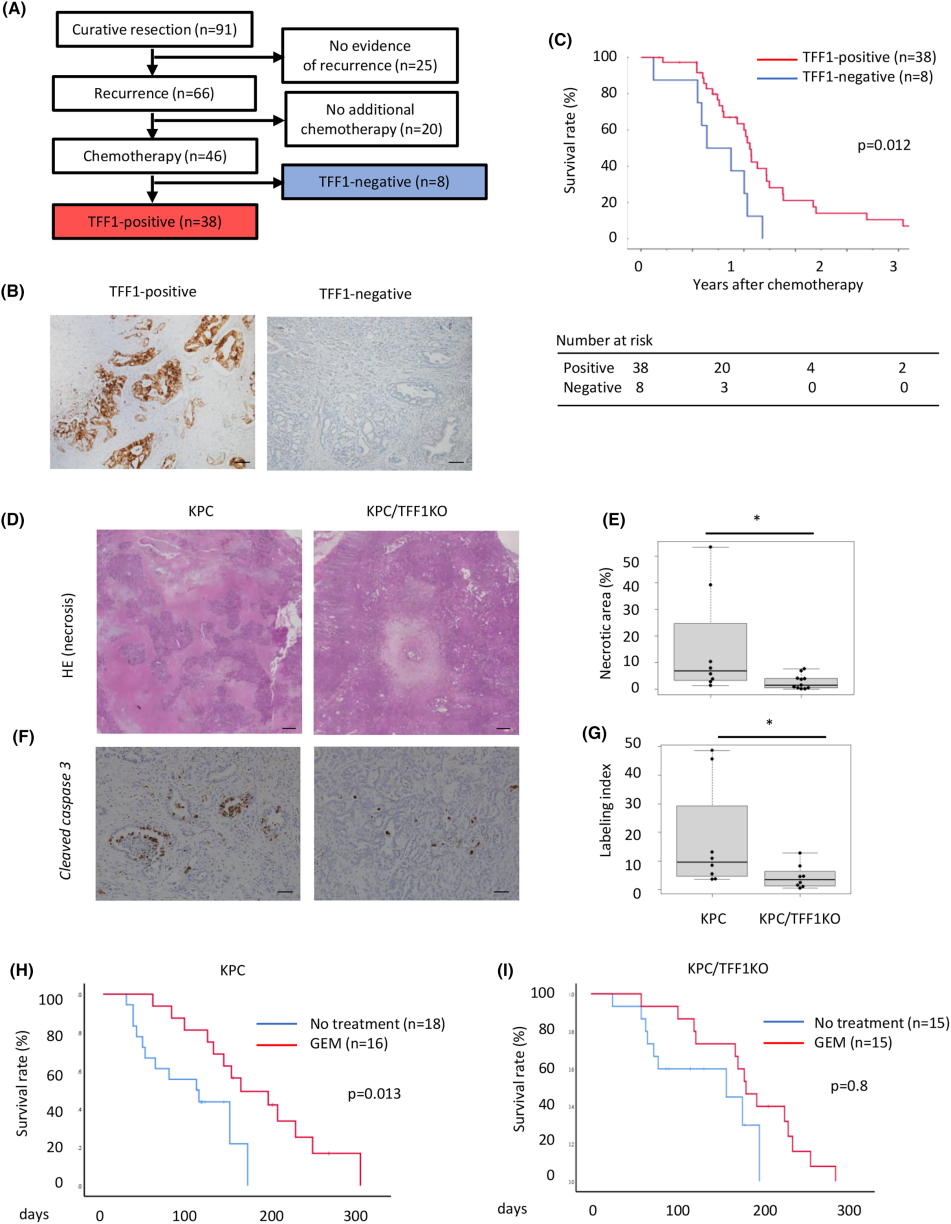
TFF1 expression enhances the chemosensitivity of pancreatic cancer in humans and mice. (A) Schema of patient selection. (B) Representative IHC images of TFF1‐positive and TFF1‐negative PDAC. Scale bars; 100 μm. (C) Overall survival of the patients after chemotherapy. (D) Representative microscopic image of PDAC found in the KPC and KPC/TFF1KO mice treated with gemcitabine. Scale bars; 200 μm. (E) Quantification of the necrotic area shown as a percentage. 19 mice (8 of KPC and 11 of KPC/TFF1KO) were investigated. *p < 0.05 (F) Representative IHC image of cleaved caspase 3 in each mouse model. Scale bars; 100 μm. (G) Quantification of cleaved caspase 3‐positive cells shown as the labelling index. 80 images from 16 mice were investigated. *p < 0.05 (H) Overall survival of the gemcitabine‐treated KPC mice. (I) Overall survival of the gemcitabine‐treated KPC/TFF1KO mice.

To investigate the chemosensitivity of human pancreatic cancer, we assessed the expression of TFF1 in surgically resected human pancreatic cancer specimens. As we have reported previously, the positivity rate of TFF1 in pancreatic cancer was almost 80%, and positivity was not associated with the survival rate after curative surgical resection.^17^ To further investigate the association of TFF1 expression and chemosensitivity, we analyzed patients who suffered postoperative disease recurrence and underwent chemotherapy (Figure 3A). The expression of TFF1 was investigated in the resected specimen (Figure 3B), revealing that the patients with positive expression of TFF1 showed a better survival rate than the patients with negative expression (Figure 3C, *p* = 0.012).

To further confirm the association of TFF1 expression and chemosensitivity, we treated the KPC and KPC/TFF1KO mice with gemcitabine. Mice were put on treatment when they were 2‐months old and gemcitabine was administered intraperitoneally with the dose of 40 mg/kg, twice/week. Pathologically, the tumors of the KPC mice were found to have higher necrotic areas than those of the KPC/TFF1KO mice (Figure 3D,E), and apoptotic cells evaluated by cleaved caspase 3 were found more frequently in the KPC mice than the KPC/TFF1KO (Figure 3F,G). When the survival of gemcitabine‐treated mice was compared to the non‐treated mice (shown in Figure 1K), the KPC mice showed improved survival when treated with gemcitabine whereas the KPC/TFF1KO mice did not (Figure 3H,I), suggesting that gemcitabine‐induced EMT^18^ was accelerated by the deficiency of TFF1 thus resulted in chemoresistance.

### 3.3 Extracellular TFF1 enhances the chemosensitivity of pancreatic cancer cells *in vitro*

**FIGURE 4:**
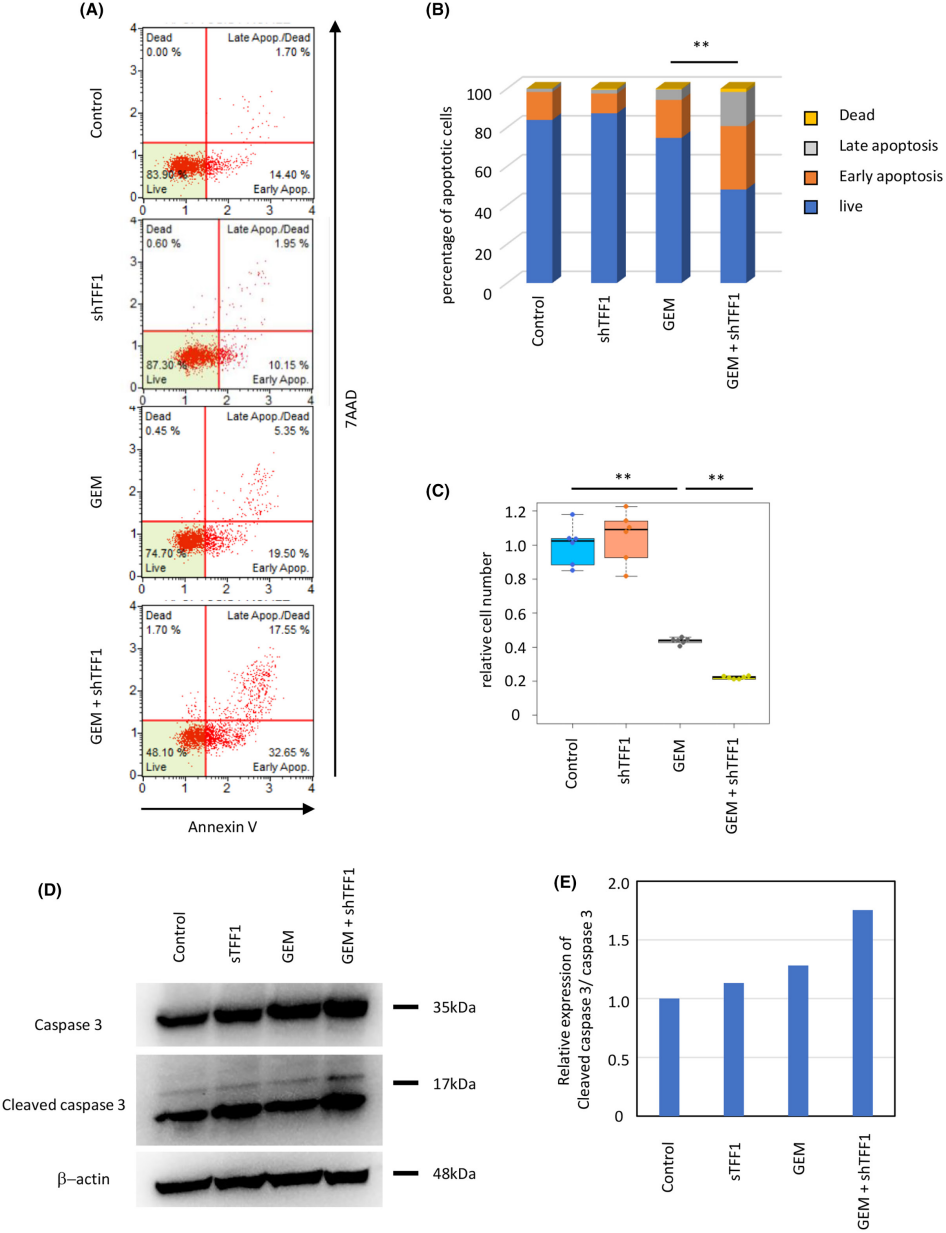
shTFF1 enhances the chemosensitivity of pancreatic cancer cells (panc1) *in vitro*. (A, B) The cells were treated with gemcitabine (1 μM) and/or shTFF1 (100 ng/mL), and then, the rate of apoptotic cells was assessed by a Muse™ Annexin V & Dead Cell Kit. **p < 0.01 (C) Relative cell number was evaluated by WST‐1 assays. **p < 0.01 (D) The expression of caspase 3 and cleaved caspase 3 in each group assessed by western blotting. (E) Quantification of the blotting shown in (D).

Although TFF1 expression in tumor cells is associated with chemosensitivity, TFF1 is a secreted protein, and it remains unclear whether TFF1 functions intracellularly or extracellularly. We hypothesized that TFF1 could display its effect even when administered extracellularly in the form of protein. To confirm this, we chemically synthesized human TFF1 peptides (hereafter referred to as shTFF1) with conserved steric structures. Panc1 cells were then treated with shTFF1 and gemcitabine to investigate their chemosensitivity. As a result, shTFF1 increased the rate of apoptotic cells induced by gemcitabine (Figure 4A,B). Interestingly, shTFF1 worked the best at the dose of 1 and 100 ng/mL (Figure S1). The relative cell number was decreased by gemcitabine, and shTFF1 enhanced this effect (Figure 4C). Cleaved caspase 3, an apoptotic marker, was also upregulated by combined treatment with gemcitabine and shTFF1 (Figure 4D,E). These results suggest that extracellularly administered shTFF1, as well as the cellular expression of TFF1, improves the chemosensitivity of pancreatic cancer cells.

### 3.4 Extracellular TFF1 inhibits gemcitabine‐induced EMT and Wnt activity *in vitro*

**FIGURE 5:**
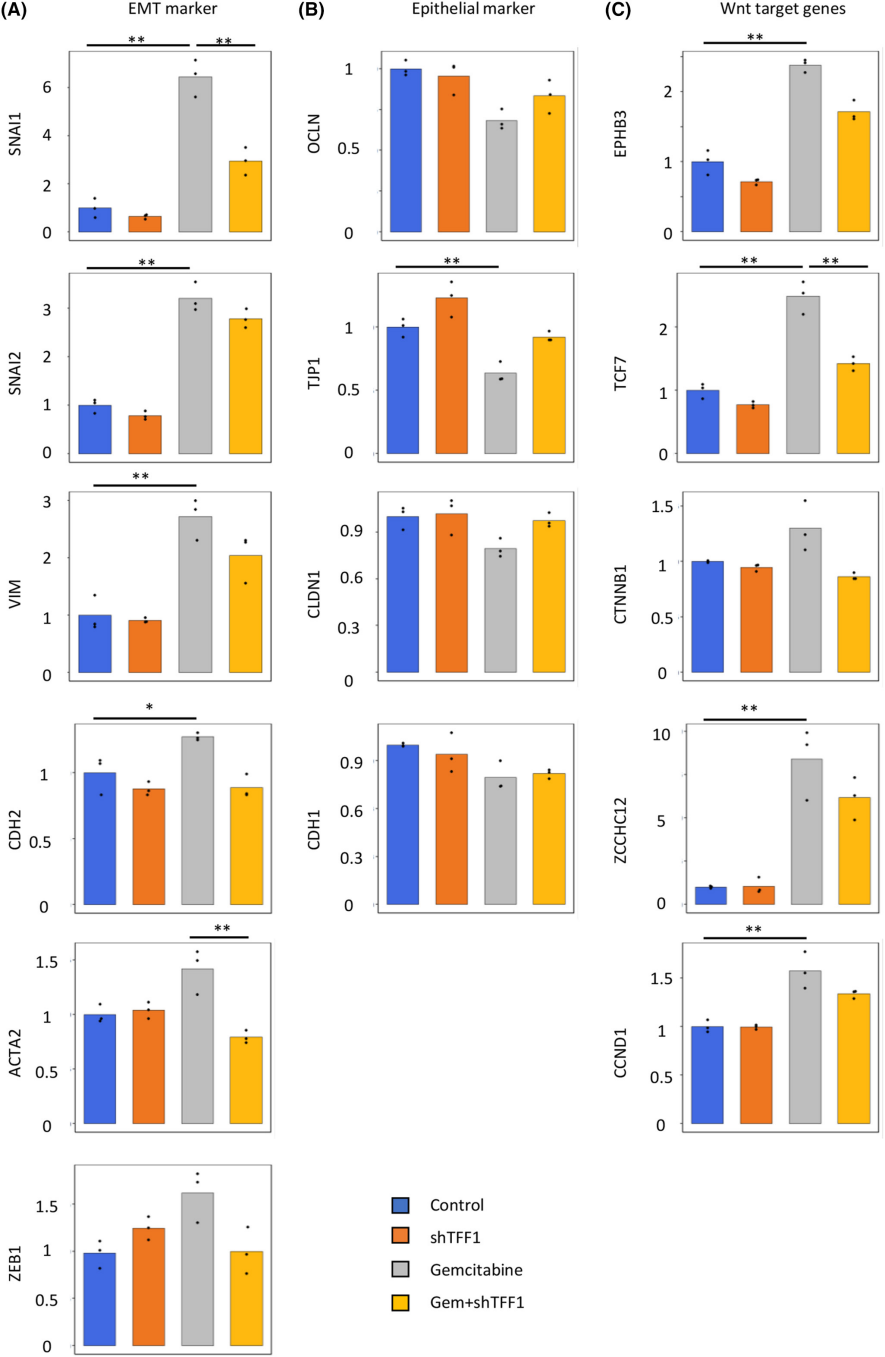
shTFF1 inhibits gemcitabine‐induced EMT and the Wnt pathway *in vitro*. The panc1cells were treated with gemcitabine (1 μM) and/or shTFF1 (100 ng/mL), and then, the relative gene expression of EMT markers (A), epithelial markers (B), and Wnt target genes (C) was assessed by RT‐PCR. *p < 0.05 and fold change>1.5, **p < 0.01 and fold change>1.5.

Clinically, repeated chemotherapy frequently results in tumor resistance, probably due to the induction of EMT in cancer cells at least in one way.^3^, ^18^ To investigate the association of gemcitabine‐induced EMT and the role of shTFF1, we treated Panc1 cells with gemcitabine and/or shTFF1, and then, their molecular characteristics were analyzed. Real‐time PCR revealed that various EMT markers (SNAI1, SNAI2, VIM, CDH2, ACTA2, and ZEB1) were upregulated by gemcitabine, and this impact was counteracted by the addition of shTFF1 (Figure 5A). In contrast, epithelial markers (OCLN, TJP1, CLDN1, and CDH1) were downregulated by gemcitabine and re‐upregulated by the addition of shTFF1 (Figure 5B). Given the previous reports revealing the association between Wnt pathway and EMT,^19^ Wnt target genes (EPHB3, TCF7, CTNNB1, ZCCHC12, and CCND1) were analyzed, revealing a similar pattern as EMT markers (Figure 5C). These results indicate that shTFF1 functions to minimize the activation of the Wnt pathway induced by gemcitabine, thus suppressing EMT and eventually inducing chemosensitivity in pancreatic cancer.

**FIGURE 6:**
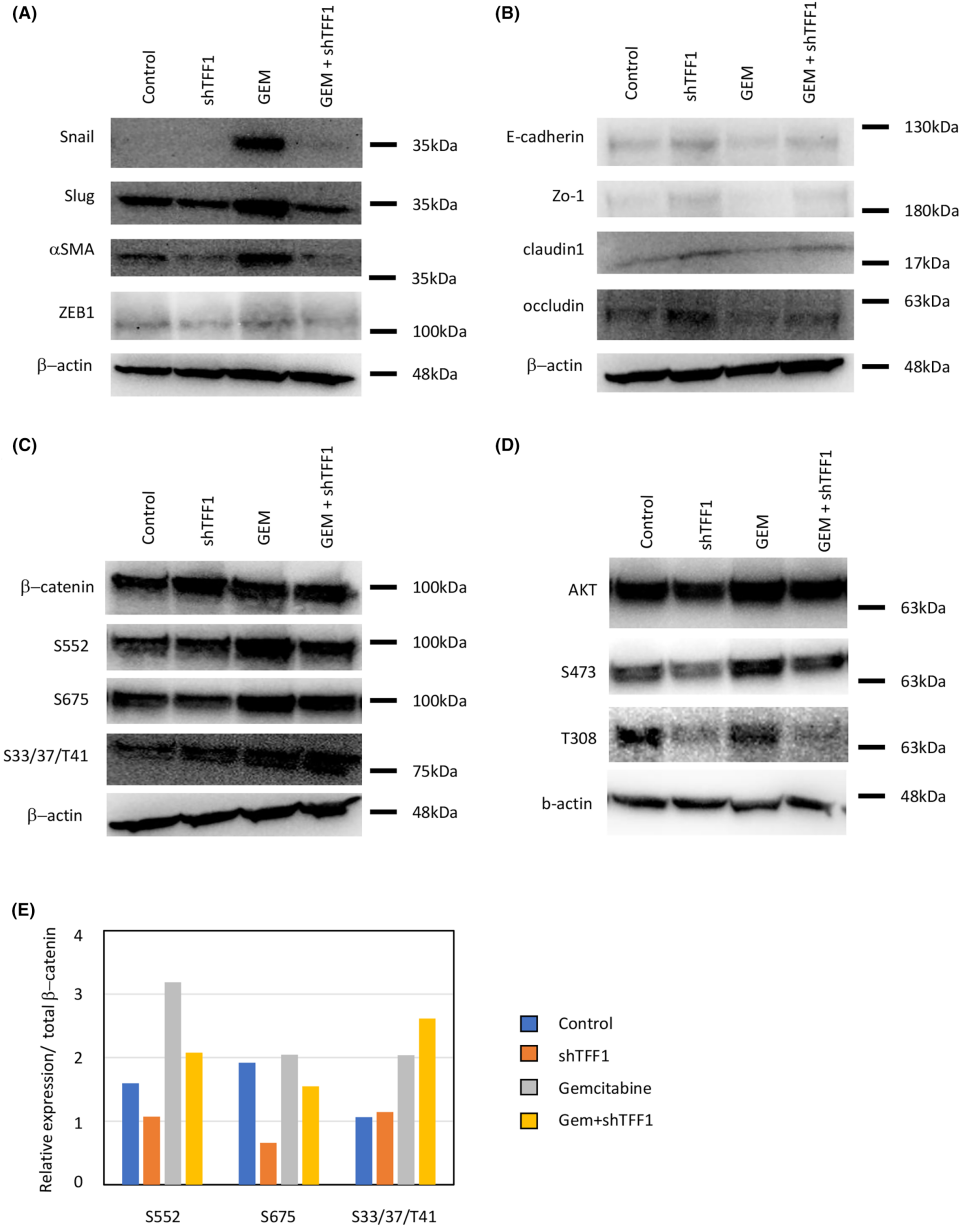
shTFF1 inhibits gemcitabine‐induced EMT and the Wnt pathway in protein level in panc1 cells *in vitro*. Protein expression levels of EMT markers (A), epithelial markers (B), β‐catenin phosphorylation (C), and AKT phosphorylation (D) were assessed by western blotting. (E) Quantification of the blotting shown in (C).

To confirm these changes in protein expression, we performed western blotting using cell lysates treated as described above. The EMT markers (Snail, Slug, αSMA, and ZEB1) showed a similar pattern, in which upregulation by gemcitabine was canceled by additional shTFF1 (Figure 6A). Conversely, epithelial markers (E‐cadherin, Zo‐1, claudin 1, and occludin) were downregulated by gemcitabine and re‐upregulated by addition of shTFF1, confirming the EMT characteristics of each group (Figure 6B). For Wnt pathway activity, the phosphorylation status of β‐catenin was investigated. Phosphorylation at Ser552 and Ser675, which are associated with the stabilization and accumulation of β‐catenin in the nucleus, was upregulated by gemcitabine and downregulated by additional shTFF1 (Figure 6C). Phosphorylation at Ser33, Ser37, and Thr41, which indicates the destabilization of β‐catenin, was increased by shTFF1 treatment. In addition, phosphorylation of AKT, the downstream effector of Wnt signaling, was decreased in the shTFF1‐treated group (Figure 6D,E). These results reconfirmed the function of shTFF1 in inhibiting EMT and Wnt signaling induced by gemcitabine treatment.

### 3.5 TFF1 inhibits cancer stemness induced by gemcitabine *in vitro*

**FIGURE 7:**
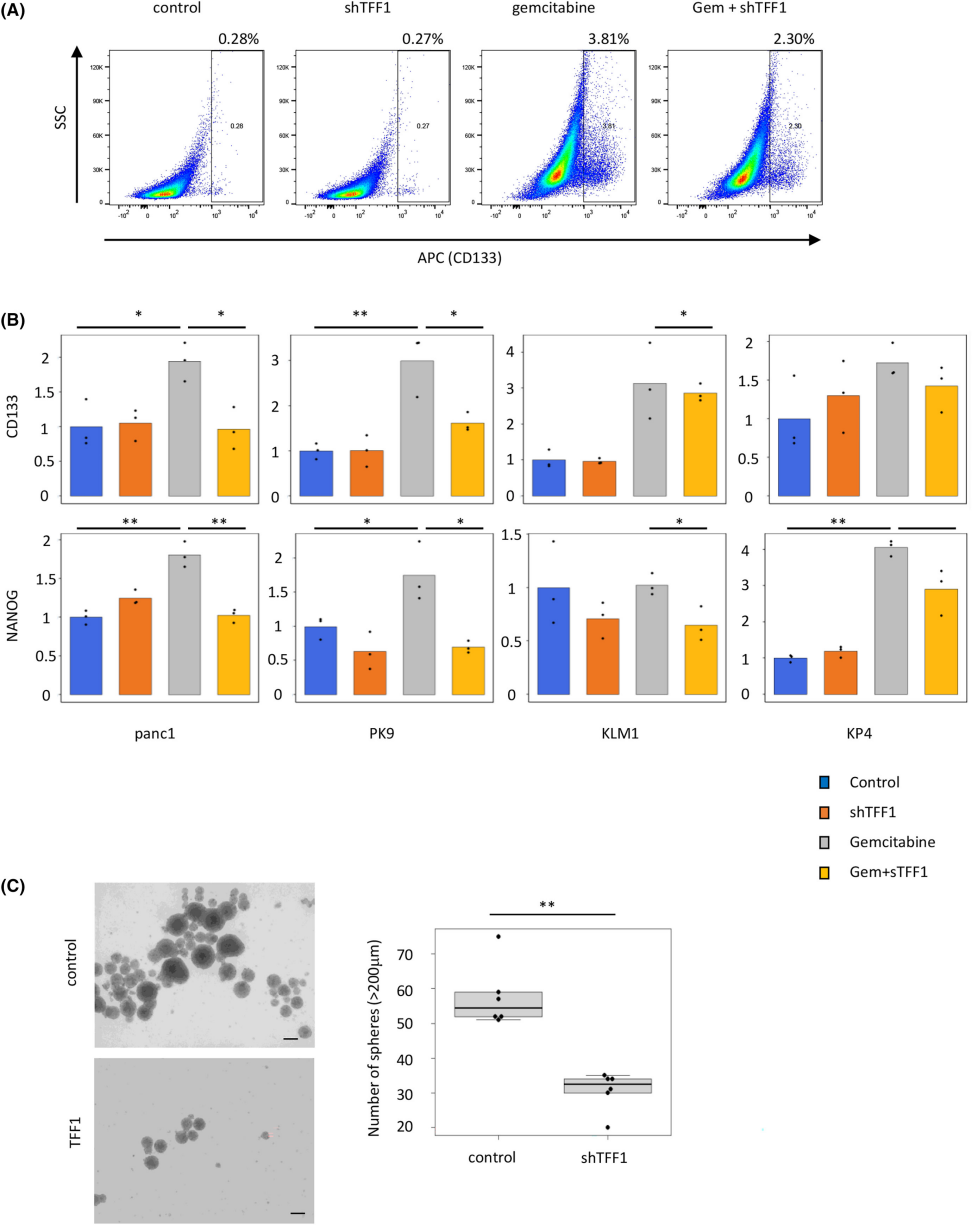
shTFF1 inhibits cancer stemness induced by gemcitabine *in vitro*. (A) Flow cytometric image of Panc1 cells treated with gemcitabine (1 μM) and/or shTFF1 (100 ng/mL). (B) The expression of cancer stem cells (CD133 and NANOG) in pancreatic cancer cells (Panc1, PK9, KLM1, and KP4) treated with gemcitabine and/or shTFF1 assessed by RT‐PCR. *p < 0.05, **p < 0.01 (C) The tumor sphere assay using KLM1. shTFF1 (100 ng/mL) suppressed the formation of tumor spheres. Scale bars; 200 μm, **p < 0.01.

We then hypothesized that the chemoresistance and EMT inhibited by TFF1 are closely associated with cancer stemness, and the stem cell markers of pancreatic cancer cell lines were investigated under gemcitabine and shTFF1 treatment. Panc1 cells were treated with gemcitabine and shTFF1, and then, the expression of CD133 was analyzed by flow cytometry. As expected, while the proportion of CD133‐positive cells was increased by gemcitabine treatment, it was decreased by the addition of shTFF1 (Figure 7A). This finding was consistent with the changes in EMT and Wnt signaling mentioned above. In terms of the cellular expression of TFF1, Panc1 cells show relatively high expression of TFF1 among various human pancreatic cancer cell lines.^11^ To explore the generality of the efficacy of shTFF1, we employed additional pancreatic cancer cell lines with relatively low TFF1 expression (PK9, KLM1, and KP4), and stem cell markers (CD133 and NANOG) were analyzed by real‐time PCR, revealing that the stemness of the cells was generally enhanced by gemcitabine treatment and counteracted by the addition of shTFF1 (Figure 7B). Next, the stemness of pancreatic cancer cells (KLM1) was investigated by tumor sphere formation assay (Figure 7C). After 14 days of incubation, the size and number were counted revealing that shTFF1 resulted in the small number of spheres significantly, also indicating that TFF1 suppress cancer stemness.

### 3.6 Combined treatment with gemcitabine and shTFF1 arrests tumor growth *in vivo*

**FIGURE 8:**
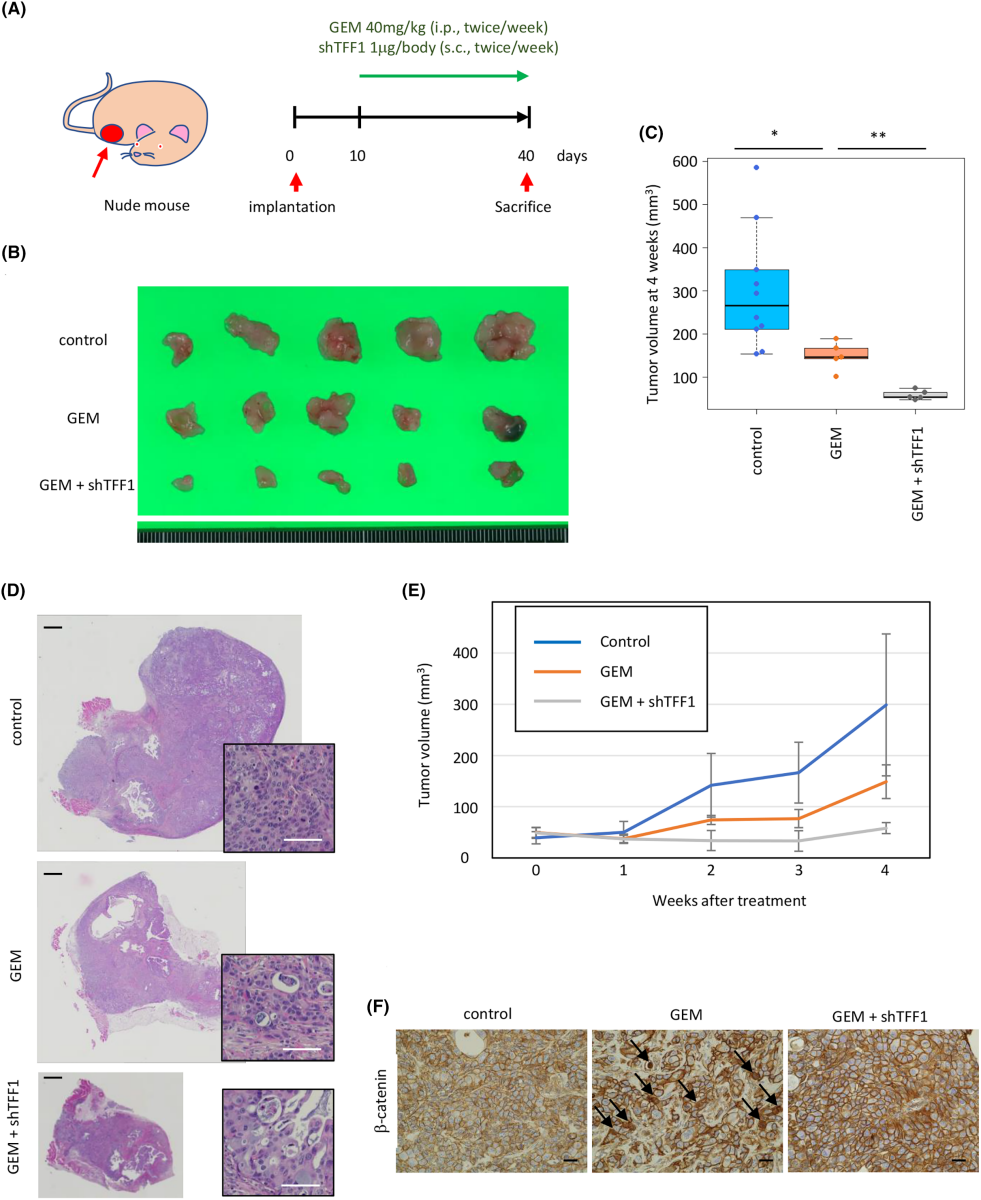
Combined treatment with gemcitabine and shTFF1 arrests tumor growth *in vivo*. (A) Schema of the schedule of mouse treatment. (B) Gross appearance of implanted tumors harvested after 4 weeks of treatment. (C) Quantification of the tumor volume in the mice treated with gemcitabine and shTFF1. *p < 0.05, **p < 0.01. (D) Representative HE image of the tumor. Scale bars; 1 mm (black) and 50 μm (white). (E) Time course of the tumor volume in each group of mice. (F) Representative IHC image of β‐catenin in each group. Scale bars; 20 μm.

To investigate the efficacy of shTFF1 *in vivo* and to explore the possible use of shTFF1 in pancreatic cancer treatment, we employed a xenograft mouse model. Panc1 cells were transplanted subcutaneously into nude mice, and the mice were treated with gemcitabine (intraperitoneally, twice per week, 40 mg/kg) and/or shTFF1 (subcutaneously, twice per week, 1 μg/body) (Figure 8A). Eventually, the tumors were harvested after 4 weeks of treatment (Figure 8B). While the volume of the tumor increased with time in the control mice, gemcitabine treatment inhibited this growth, and the additional shTFF1 significantly accelerated the efficacy of gemcitabine (Figure 8C). The HE image of the tumors confirmed the existence of adenocarcinoma (Figure 8D). Of note, combined treatment with gemcitabine and shTFF1 almost arrested the growth of the tumor (Figure 8E), supporting the possibility of combined treatment with TFF1 and traditional chemotherapy. The expression of β‐catenin was investigated by IHC, revealing the nuclear translocation of β‐catenin in gemcitabine‐treated tumors which disappeared in both‐treated tumors (Figure 8F), confirming the Wnt‐suppressing role of shTFF1.

### 3.7 TFF1 has an effect on spontaneously developed pancreatic cancer

**FIGURE 9:**
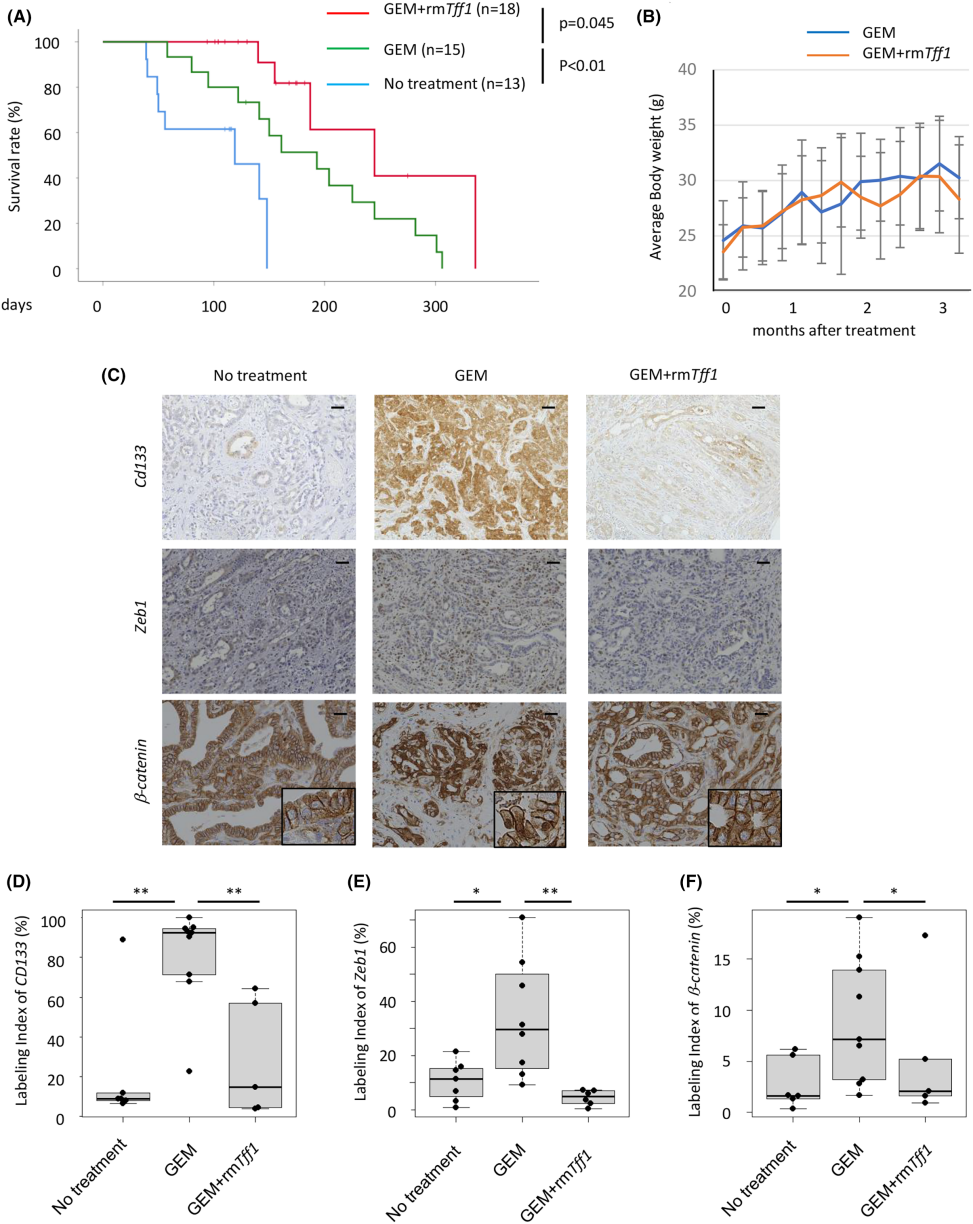
Recombinant TFF1 enhances the chemosensitivity of pancreatic cancer in mice. (A) Disease‐specific survival of the KPC mice treated with gemcitabine and rmTFF1. (B) Average body weight of each group of mice treated with gemcitabine and/or rmTFF1. (C) Representative IHC image of CD133, ZEB1 and β‐catenin in mouse tumors. Scale bars; 100 μm. (D) Quantification of CD133 positive cells shown as a labelling index. (E) Quantification of ZEB1‐positive cells shown as a labelling index. (F) Quantification of nuclear β‐catenin positive cells shown as a labelling index. *p < 0.05, **p < 0.01.

To further investigate the impact of gemcitabine/TFF1 treatment *in vivo*, we administered this treatment to a KPC mouse model of pancreatic cancer. In the absence of synthesized mouse TFF1, we employed recombinant mouse *Tff1* (rm*Tff1*) instead. Gemcitabine and *Tff1* were administered with the same protocol as in the xenograft model, starting at the age of 2 months old. As expected, gemcitabine alone improved the disease‐specific survival of the KPC mice, and rm *Tff1* further improved this survival time (Figure 9A). The mice treated with rm *Tff1* did not show any signs of adverse events, and the body weight increased, similar to that in the gemcitabine alone group, suggesting that the additional *Tff1* treatment was well tolerated (Figure 9B). The mice were harvested when they showed any sign of morbidity, and the tumors of these mice were analyzed by immunohistochemistry, revealing that *Cd133*, *Zeb1* expression and nuclear translocation of *β‐catenin* was increased in the gemcitabine group but re‐downregulated in the gemcitabine/rm *Tff1* group (Figure 9C–F). These results confirmed that the impact of *Tff1* on EMT and stemness was reproducible in this mouse model. These results suggest the efficacy of gemcitabine/*Tff1* treatment for pancreatic cancer, not only in the implantation model but also in the spontaneous cancer‐developing model in which the tumor microenvironment is preserved.

## 4 DISCUSSION

In this study, we revealed that TFF1 acts as a tumor suppressor to inhibit EMT and cancer stemness, thus enhancing the chemosensitivity of pancreatic cancer. Importantly, extracellularly administered TFF1 exhibit a similar function as cellular expression of TFF1, indicating that TFF1 therapy might be a promising approach for pancreatic cancer treatment in the near future.

TFFs are secreted proteins predominantly expressed in the gastrointestinal mucosal epithelium as a factor with mitogenic activity.^20^ We have investigated their functions in the pancreas and liver, revealing that TFFs are expressed in the stem or progenitor cell niche^21^, ^22^ and responsible for regeneration and carcinogenesis in these organs.^11^, ^12^, ^17^, ^23^, ^24^, ^25^ It is suggested that loss of control of proliferation and differentiation in the stem cell niche eventually causes the development of neoplastic diseases.^26^, ^27^ Admittedly, the loss of TFF2 resulted in the development of pancreatic neoplasms.^25^ Considering these evidences, it seems plausible that TFF1 is closely associated with the stemness of neoplastic cells, and it is consistent with the finding of this study that TFF1 inhibits stemness of pancreatic cancer.

TFFs are secreted into the gastrointestinal lumen with mucins to protect the mucosal epithelium,^28^ whereas TFFs also exist in the bloodstream and urine,^29^, ^30^ suggesting that TFFs might function not only in a paracrine or autocrine fashion but also in a hormonal fashion, and this study revealed that subcutaneously administered TFF1 significantly suppressed tumor growth *in vivo*. Nevertheless, it remains to be clarified how secreted TFF1 functions in distant organs. One hypothesis is that TFF1 might act as a ligand to interact with a receptor of the cells. In fact, previous reports suggest that TFF1 bind as a lectin to a hypothetical glycosylated receptor.^31^ Similar to secreted frizzled‐related proteins (SFRPs), which act as antagonists to the Wnt receptor frizzled (FZD),^32^, ^33^ TFF1 may function in a same way to inhibit Wnt signaling pathway. Nevertheless, further investigations are needed to clarify the precise mechanisms of the tumor‐suppressive role of TFF1.

This study clearly showed the efficacy of TFF1, if treated with gemcitabine, in inhibiting the tumor growth of pancreatic cancer in a xenograft model, suggesting that TFF1 might be useful to develop novel treatment for pancreatic cancer. On the other hand, TFF1 also can be useful for the prevention of disease development. Given that deficiency of TFF1 in KC mice (the model with premalignant lesion) resulted in the development of pancreatic cancer,^11^ TFF1 functions to inhibit the initiation of malignant disease. Some populations, such as those with chronic pancreatitis (for pancreatic cancer) and liver cirrhosis (for hepatocellular carcinoma), have a high risk of cancer development. To date, there is no effective treatment for these patients to prevent cancer development. Once preventive therapy by TFF1 is established, it might allow these patients to avoid future surgical treatment and chemotherapy.

In conclusion, we found that TFF1 inhibits EMT and stemness in pancreatic cancer. The chemosensitivity of the tumor was increased by injected TFF1; thus, combined therapy with TFF1 and traditional chemotherapy could be useful in pancreatic cancer treatment. Further experiments are needed to reveal the precise mechanisms of TFF1 as a tumor suppressor in various organs.

## AUTHOR CONTRIBUTIONS

**Junpei Yamaguchi:** Conceptualization (lead); funding acquisition (lead); investigation (lead); methodology (lead); visualization (lead); writing – original draft (lead). **Toshio Kokuryo:** Supervision (lead). **Yukihiro Yokoyama:** Supervision (supporting). **Shunsuke Oishi:** Resources (equal). **Masaki Sunagawa:** Data curation (supporting). **Takashi Mizuno:** Data curation (supporting). **Shunsuke Onoe:** Data curation (supporting). **Nobuyuki Watanabe:** Data curation (supporting). **Atsushi Ogura:** Data curation (supporting). **Tomoki Ebata:** Data curation (supporting); project administration (supporting).

## FUNDING INFORMATION

This work was supported by the Japan Society for the Promotion of Science (JSPS) (JSPS KAKENHI grant numbers 17K10695 and 20H03751).

## CONFLICT OF INTEREST STATEMENT

The authors have no conflict of interest.

## ETHICS STATEMENT

Human samples were collected and analyzed in accordance with approval from the Institutional Review Board of Nagoya University (approved number; 2017‐0080). The informed consent was obtained from all patients. All animal experiments were conducted in accordance with approval from the Institute for Laboratory Animal Research, Nagoya University Graduate School of Medicine (approved number; 20328).

## Supporting information


Figure S1.

Table S1.
Table S2.
Table S3.

## Data Availability

All data generated and analysed during the current study are included in this article and supplementary information files. The additional data that support the findings of this study are available from the corresponding author on reasonable request.
