# Supplementary figures and images for: Trefoil factor 1 suppresses stemness and enhances chemosensitivity of pancreatic cancer
Source: Cancer Med. 2024 Jun 13;13(11):e7395. doi: 10.1002/cam4.7395 (PMC11176577; doi:10.1002/cam4.7395)

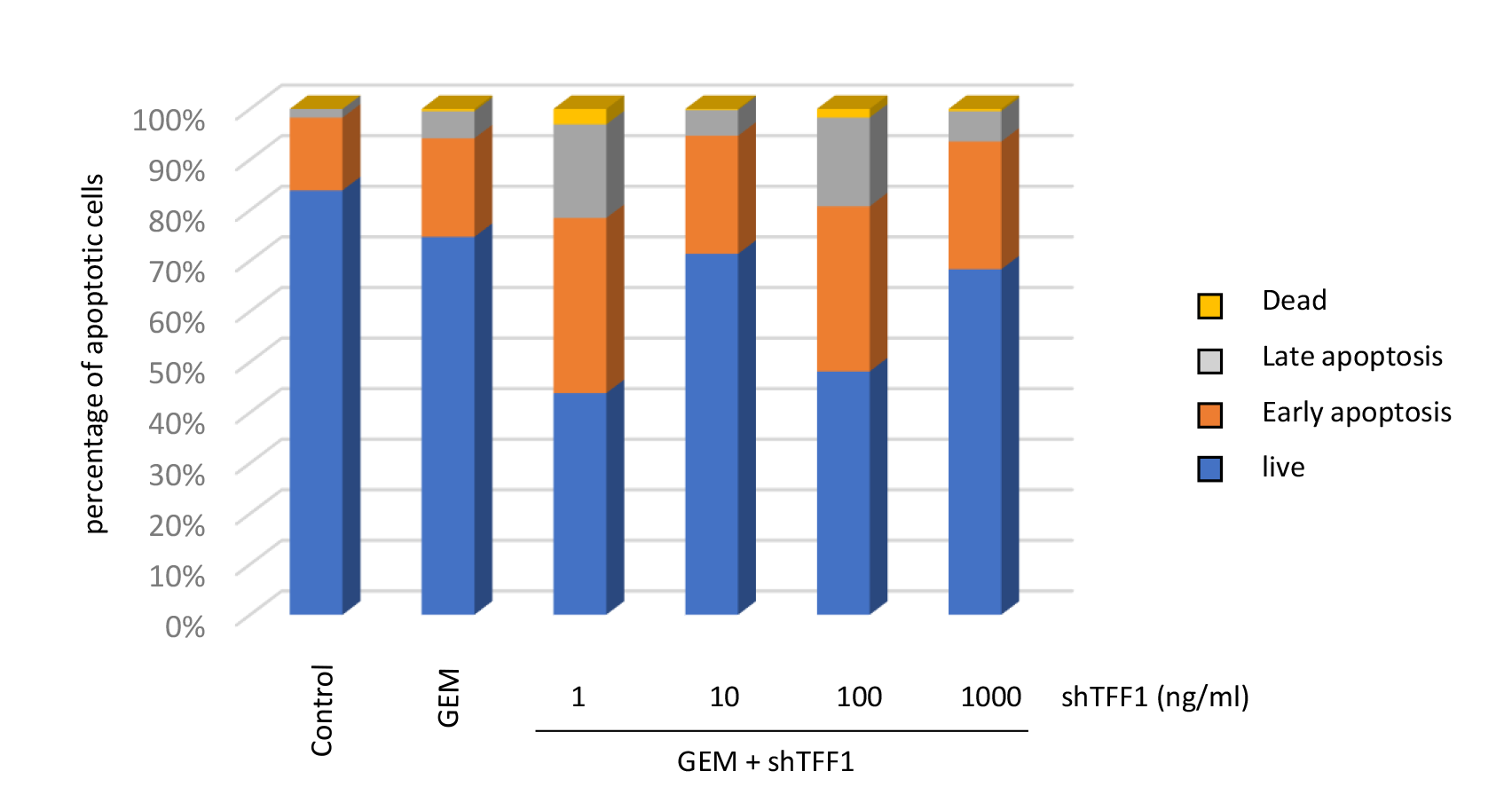

Supplement: Supplementary file 1 — Figure S1. [file CAM4-13-e7395-s002.tif]
