# Supplementary material for: Trefoil factor 1 suppresses stemness and enhances chemosensitivity of pancreatic cancer
Source: Cancer Med. 2024 Jun 13;13(11):e7395. doi: 10.1002/cam4.7395 (PMC11176577; doi:10.1002/cam4.7395)
Supplement: Supplementary file 2 — Table S1. Table S2. Table S3. [file CAM4-13-e7395-s001.docx]

Supplemental Table 1. Conditions and primary antibodies for immunohistochemistry.

| protein of interest | antigen | antibody by | clone/catalog# | concentration |
| --- | --- | --- | --- | --- |
| TFF1 | human | ThermoFisher | GE2 | 1:50 |
| TFF1 | mouse | origene | TA322883 | 1:50 |
| Snail | mouse | abcam | ab180714 | 1:500 |
| Ki67 | mouse | abcam | ab16667 | 1:200 |
| ZEB1 | mouse | NOVUS biologicals | NBP1-05987 | 1:250 |
| cleaved caspase 3 | mouse | abcam | D175 | 1:200 |
| CD133 | mouse | abcam | ab19898 | 1:200 |
| β-catenin | Human,  mouse | BD Transduction | 610154 | 1:400 |
| CK19 | mouse | DSHB* | TROMAIII | 1:100 |

* DSHB: Developmental Studies Hybridoma Bank

Supplemental Table 2. Conditions and primary antibodies for western blotting.

| protein of interest | antibody by | clone/catalog# | concentration |
| --- | --- | --- | --- |
| TFF1 | origene | TA322883 | 1:1000 |
| snail | CST | ab180714 | 1:1000 |
| slug | CST | C1967 | 1:1000 |
| caspase 3 | CST | 9662 | 1:1000 |
| cleaved caspase 3 | CST | D175 | 1:1000 |
| caspase 7 | CST | 9492 | 1:1000 |
| cleaved caspase 7 | CST | D198 | 1:1000 |
| αSMA | abcam | ab5694 | 1:1000 |
| ZEB1 | NOVUS biologicals | NBP1-05987 | 1:1000 |
| E-cadherin | CST | 2.40E+11 | 1:1000 |
| Zo-1 | CST | D7012 | 1:1000 |
| claudin-1 | CST | D5H1D | 1:1000 |
| Occludin | CST | ab168986 | 1:1000 |
| βcatenin | BD transduction | 610154 | 1:1000 |
| phospho-βcatenin (S552) | CST | D8E11 | 1:1000 |
| phospho-βcatenin (S675) | CST | D2F1 | 1:1000 |
| phospho-βcatenin (S33/37/T41) | CST | 9561T | 1:1000 |
| CD133 | abcam | ab19898 | 1:1000 |
| AKT | CST | 9272 | 1:1000 |
| phospho-AKT (S473) | CST | 9271 | 1:1000 |
| phospho-AKT (T308) | CST | 9275 | 1:1000 |
| β-actin | Sigma-Aldrich |  | 1:2000 |

Supplemental Table 3. Primers for RT-PCR

| gene of interest | primers by | catalog # |
| --- | --- | --- |
| SNAI1 | Applied Biosystems | Hs00195591_m1 |
| SNAI2 | Applied Biosystems | Hs00950344_m1 |
| VIM | Applied Biosystems | Hs00958111_m1 |
| CDH1 | Applied Biosystems | Hs01023895_m1 |
| CDH2 | Applied Biosystems | Hs00983056_m1 |
| ACTA2 | Applied Biosystems | Hs00426835_g1 |
| ZEB1 | Applied Biosystems | Hs01566410_m1 |
| OCLN | Applied Biosystems | Hs00170162_m1 |
| TJP1 | Applied Biosystems | Hs01551861_m1 |
| CLDN1 | Applied Biosystems | Hs00221623_m1 |
| EPHB3 | Applied Biosystems | Hs00177903_m1 |
| TCF7 | Applied Biosystems | Hs01556515_m1 |
| CTNNB1 | Applied Biosystems | Hs00355045_m1 |
| ZCCHC12 | Applied Biosystems | Hs00381614_m1 |
| CCND1 | Applied Biosystems | Hs00765553_m1 |
| NANOG | Applied Biosystems | Hs02387400_g1 |
| CD133 | Applied Biosystems | Hs10009259_s1 |
| 18S | Applied Biosystems | Hs99999901_s1 |
